# Identification of molecular subtypes and prognostic signature for hepatocellular carcinoma based on genes associated with homologous recombination deficiency

**DOI:** 10.1038/s41598-021-03432-3

**Published:** 2021-12-15

**Authors:** Hongsheng Lin, Yangyi Xie, Yinzhi Kong, Li Yang, Mingfen Li

**Affiliations:** 1 Guangxi University of Chinese Medicine, Nanning, 530200 China; 2 Department of Laboratory, The First Affiliated Hospital of Guangxi University of Chinese Medicine, Nanning, 530023 China; 3 Guangxi Medical University, Nanning, 530021 China; 4 Department of Microbiology, School of Basic Medical Sciences, Guangxi Medical University, Nanning, 530021 China; 5 The First Clinical Faculty of Guangxi University of Chinese Medicine, Nanning, 530200 China

**Keywords:** Cancer, Computational biology and bioinformatics, Biomarkers, Risk factors

## Abstract

Hepatocellular carcinoma (HCC) is a rapidly developing digestive tract carcinoma. The prognosis of patients and side effects caused by clinical treatment should be better improved. Nonnegative matrix factorization (NMF) clustering was performed using 109 homologous recombination deficiency (HRD)-related of HCC genes from The Cancer Genome Atlas (TCGA) database. Limma was applied to analyze subtype differences. Immune scores and clinical characteristics of different subtypes were compared. An HRD signature were built with least absolute shrinkage operator (LASSO) and multivariate Cox analysis. Performance of the signature system was then assessed by Kaplan–Meier curves and receiver operating characteristic (ROC) curves. We identified two molecular subtypes (C1 and C2), with C2 showing a significantly better prognosis than C1. C1 contained 3623 differentially expressed genes. A 4-gene prognostic signature for HCC was established, and showed a high predicting accuracy in validation sets, entire TCGA data set, HCCDB18 and GSE14520 queues. Moreover, the risk score was validated as an independent prognostic marker for HCC. Our research identified two molecular subtypes of HCC, and proposed a novel scoring system for evaluating the prognosis of HCC in clinical practice.

## Introduction

Liver cancer is one of the most rapidly developing digestive tract tumors^1^. Hepatocellular carcinoma (HCC), which accounts for 90% of all liver cancer types, is characterized by high mortality and poor prognosis^2^.Only 5% to 15% of HCC tumors can be surgically removed after diagnosis. The first-line treatment options for the late stage is oral dosing with sorafenib, however, but it cannot effectively improve the condition of HCC^3^. Hence, improving the prognosis of patients and reducing side effects are currently the major problems to be solved in clinical practice.

Studies have shown that homologous recombination deficiency (HRD) is common in cancer development^4^. HRD as a functional defect in homologous recombinant DNA repair could result in permanent alteration in the genome in a specific, quantifiable pattern ("genomic scar")^5^. Studies showed that HRD occurs at different frequencies in many cancer types^6^. In cancer cells with HRD, double-stranded DNA breaks are repaired through error-prone pathways (i.e., non-homologous end ligations), leading to cell death and tumor shrinkage^7,8^. Previous reports analyzed the role of HRD in a variety of cancers, and found that HRD is visibly associated with survival of patients with ovarian cancer and glioblastoma polymordia, and is also related to a poor prognosis of adrenal cortical carcinoma, squamous cell carcinoma of head and neck, clear cell carcinoma of kidney, renal papillary cell carcinoma, sarcoma and uterine corpus endometrial carcinoma^6^. However, the relationship between HRD and HCC prognosis has not been fully characterized.

As HCC has highly heterogeneous genomic aberrations and microenvironments, frequent recurrence is another challenge in the treatment of HCC^2^. Therefore how targeted therapies to kill individual cells remains a major tackle to be resolved, which also points to the clinical importance of classifying patients into relative subtypes based on key characteristics^9^. A large-scale study identified three HCC subtypes using combined data from five platforms (DNA copy number, DNA methylation, mRNA expression, miRNA expression and RPPA) and simultaneous unsupervised clustering^10^. Although many genome-wide analyses of HCC have been performed, there is still a lack of hierarchical clustering analysis of HRD-associated genes to explore the prognostic characteristics of HCC.

In this study, we performed NMF clustering to classify HCC based on HRD-related genes, and investigated the relationship between subtypes, HCC immune infiltration and clinical characteristics. A risk score model was built to predict the prognosis of HCC patients. It is hoped that the current findings will provide new insights into personalized treatment of HCC.

## Results

### Characteristics of immune infiltration in HCC

**Figure 1:**
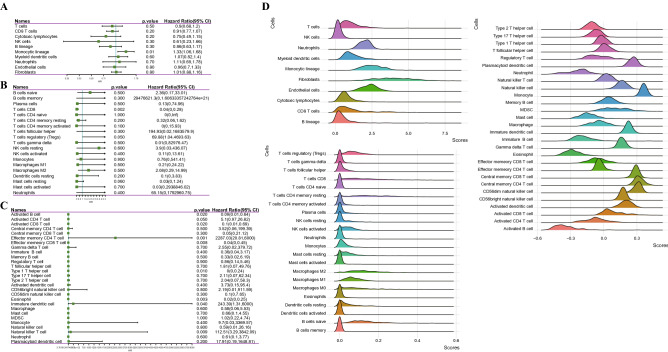
Characteristics of immune infiltration in HCC. (**A**) Univariate Cox regression analysis of MCP counter detection of immune cells and prognosis. (**B**) The forest map showed the correlation between immune cells analyzed in CIBERSORT and prognosis. (**C**) Univariate Cox regression analysis of immune cells in ssGSEA and prognosis. The mountain map shows the scores of different immune cells in HCC. (**D**) The mountain diagram showed the proportion of immune cells in HCC.

To explore the immune cell infiltration in HCC, the relationship between prognosis and immune cells was analyzed according to MCP Counter, CIBERSORT and ssGSEA. The forest map showed that CD8 T cells, monocytic lineage, activated B cells, activated CD8 T cells, effector memory CD4 T cell, type 1 T helper cell, eosinophil, immature dendritic cell and natural killer T cell played a significant role in the prognosis of HCC (Fig. 1A-C). Analysis of the proportion of each immune cell in HCC demonstrated that T cells, Neutrophils, myeloid dendritic cells, monocytic lineage, fibroblasts and endothelial cells showed a high proportion in HCC (Fig. 1D).

### Two molecular subtypes of HCC were identified by NMF clustering

**Figure 2:**
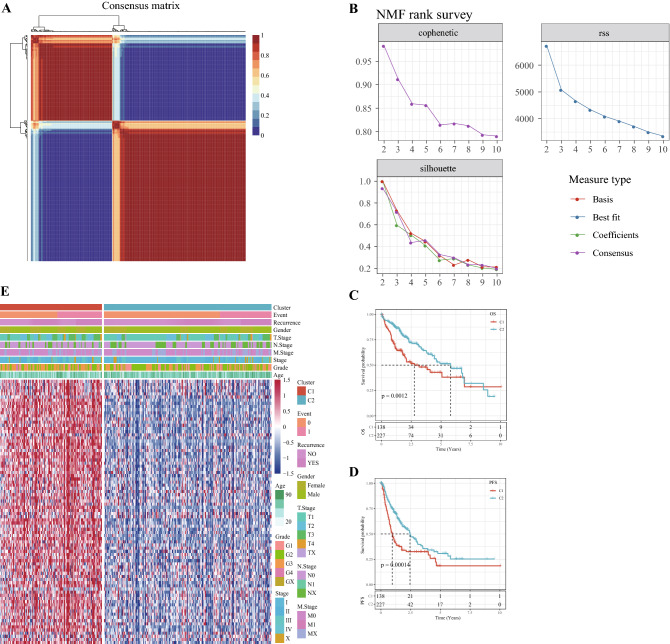
Two molecular subtypes of HCC were identified by NMF clustering. (**A**) Consensus map for NMF Clustering. (**B**) When rank = 2–10, the cophenetic correlation, residual sum of squares (RSS) and silhouette distribution. (**C**) Survival curves of C1 and C2 subtypes in TCGA dataset. (**D**) PFS of C1 and C2 subtypes in the TCGA cohort. E: Heat map of expression of 84 HRD genes in subtypes.

From 109 HRD genes, 84 genes with strong prognostic significance for HCC were screened by univariate COX analysis. NMF clustering was performed on the 84 genes, and according to cophenetic, suspension and silhouette indicators, the optimal k was determined to be 2 (Fig. 2A,B). The two molecular subtypes were defined as C1 and C2. The OS and progress-free survival (PFS) between C1 and C2 were significantly different, and OS and PFS in C2 were significantly longer than C1 (Fig. 2C,D). The gene expression heat map of the two molecular subtypes demonstrated that prognosis-related HRD genes were high-expressed in the C1 subtype (Fig. 2E).

### Identification of DEGs between subtypes and enrichment analysis

**Figure 3:**
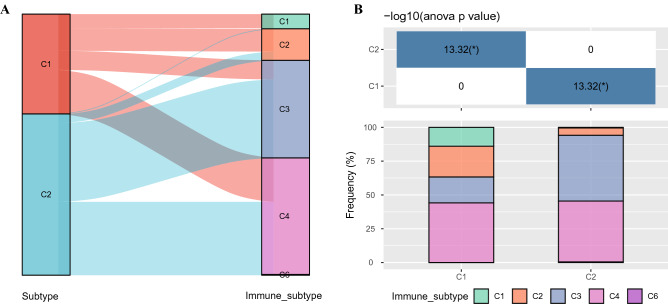
Relationship between molecular subtypes and published immune molecular typing. (**A**, **B**) Sankey graph and histogram shows the distribution of BLCA in our molecular subtypes of C1, C2, C3, C4, C5, and C6.

**Figure 4:**
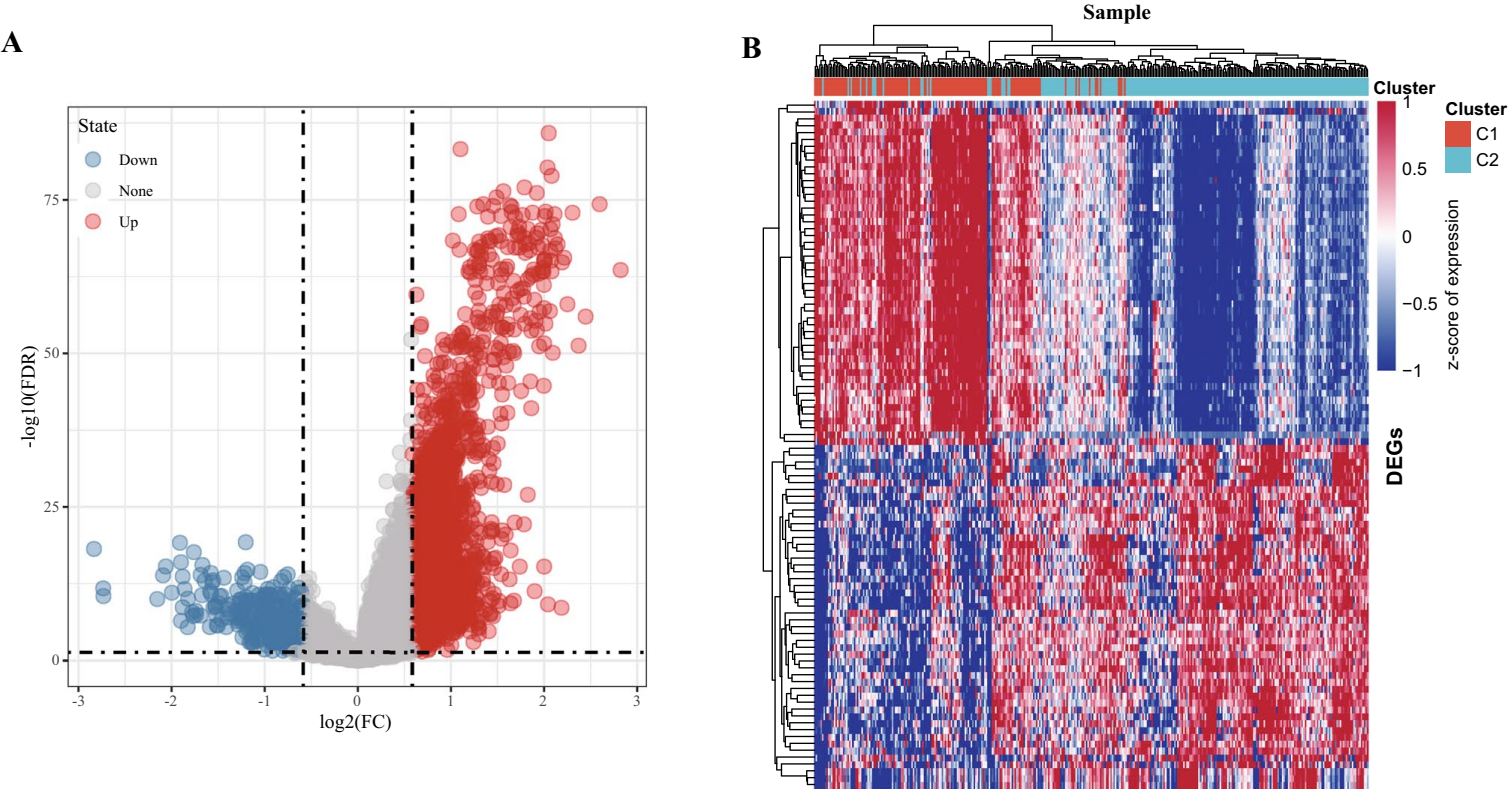
Differential expression analysis between subtypes. (**A**) DEGs between C1 and C2 molecular subtypes, the red dots represent differentially down-regulated genes and the blue dots represent differentially up-regulated genes. (**B**) Heat map of DEGs between C1 and C2 subtypes, red represents high expression DEGs, blue represents low expression DEGs, and the darker the color, the more significant it is.

**Figure 5:**
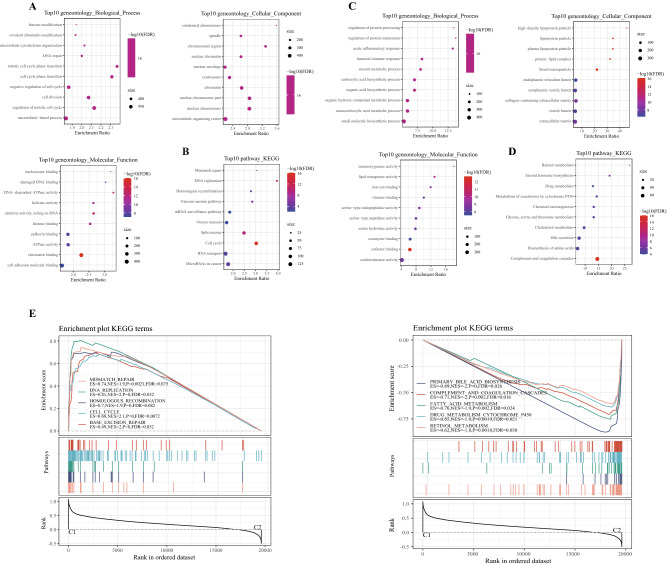
The pathway enrichment analysis of DEGS and subtypes. (**A**) Go functional annotation of differentially upregulated genes, including top 10 biological process (BP), top10 cellular component (CC) and top 10 molecular function (MF). (**B**) KEGG annotation of differentially upregulated genes. (**C**) GO functional annotation of differentially down-regulated genes. (**D**) KEGG annotation results of differentially regulated genes. E: GSEA analysis of main enriched pathways in the C1(left) and C2(right).

We also conducted association analysis between the typing results and published immunotyping results^11^ from the TCGA cohort. The results indicated that C1 subtype mainly included the wound healing(C1), IFN-γ dominant (C2), inflammatory (C3) and Lymphocyte (C4) subtypes identified by Vesteinn Thorsson et al., and that C2 subtypes mainly include C2, C3 and C4 subtypes defined by Vesteinn Thorsson (Fig. 3A,B). Difference analysis (at the threshold of FDR < 0.05 and | | FC > 1.5) showed that a total of 3623 genes were differentially expressed in C1 in comparison to C2 (Supplementary Table S1). Specifically, among the 3623 genes, 3,301 were upward DEGS and 322 were downward DEGS (Fig. 4A). The heat map of top 100 DEGs were shown in Fig. 4B. GO analysis of up-regulated DEGs demonstrated that histone modification and covalent chromatin modification (biological processes, BP), condensed chromosome and spindle (cellular components, CC), nucleosome binding and damaged DNA binding (molecular function, MF) were the most significantly enriched terms (Fig. 5A). The most prominently enriched GO terms in down-regulated DEGs were regulation of protein processing and regulation of protein maturation (BP), high-density lipoprotein particle and lipoprotein particle (CC), monooxygenase activity and lipid transporter activity (MF) (Fig. 5C). KEGG analysis showed that the main enrichment pathways for upregulation of DEGs were mismatch repair, DNA replication, and homologous recombination (Fig. 5B). Supplementary S1.txt showed other up-regulated DEGs significantly enriched in GO terms and KEGG pathway in addition to Fig. 3A and B. The down-regulated DEGs were significantly enriched in metabolism-related pathways, including retinol metabolism, steroid hormone biosynthesis and drug metabolism (Fig. 5D). Supplementary S2.txt showed down-regulated DEGs significantly enriched in GO terms and KEGG pathway in addition to Fig. 3C and D mentioned. Moreover, GSEA also identified significant enrichment pathways for the C1 subtype, including mismatch repair, DNA replication, homologous recombination, cell cycle and base excision repair, while the C2 subtype was related to fatty acid metabolism, complement and coagulation cascades, retinol metabolism, drug metabolism cytochrome p450, and retinol metabolism (Fig. 5E). Therefore, the C1 subtype was more associated with tumor development, while the C2 subtype was more associated with metabolism.

### Relationship between molecular subtypes, immune infiltration and clinical features

**Figure 6:**
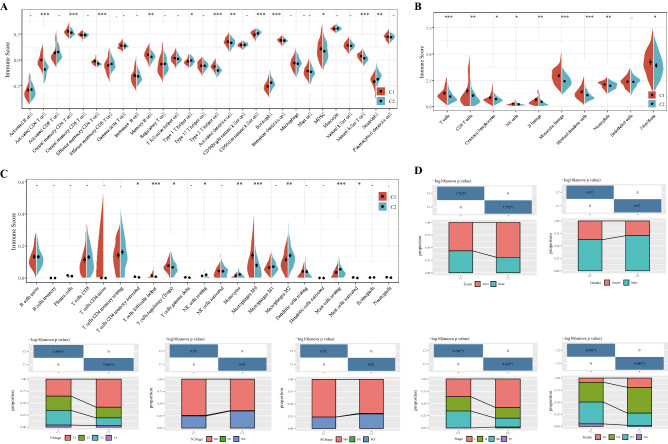
Relationship between molecular subtypes and immune infiltration and clinical features. (**A**) Immune scores of 28 immune cells in C1 and C2 subtypes were analyzed by ssGSEA. (**B**) MCP Counter calculated the immune scores of 10 types of immune cells between two clusters. (**C**) Immune scores of 22 infiltrative immune cell components in C1 and C1 subtypes of tumors. (**D**) Event, gender, T stage, N stage, M stage, AJCC stage and grade distribution of each subtype in the TCGA cohort.

Immune score reflects immune infiltration based on lymphocyte gene expression^12^. Hence, the immune score was applied to estimate the state of immune cell infiltration in HCC samples. The C1 subtype exhibited a significantly higher immune score of activated CD4 T cell, central memory CD4 T cell, effector memory CD4 T cell, memory B cell, type 2 T helper cell, activated dendritic cell, myeloid-derived suppressor cell (MDSC) and natural killer T cell (Fig. 6A). The analysis results of MCP counter demonstrated that T cells, CD8 T cells, cytotoxic lymphocytes, NK cells, B lineage, monocytic lineage, myeloid dendritic cells, neutrophils, fibroblasts obtained significantly high immune scores in C1 subtype of HCC (Fig. 6B). ESTIMATE was used to evaluate the infiltration status of immune cells in HCC subtype of TCGA. Compared with C2 subtype, the infiltration degree of activated memory T cells, CD4 follicular helper T cells, regulatory T cells (Tregs), M0 macrophages and activated mast cells in C1 subtype was significantly higher, while the infiltration degree of resting NK cells, monocytes, M2 macrophages and resting mast cells was sharply lower (Fig. 6C). These results suggested that tumors from different subtypes showed considerable variation in their immunoinfiltrative status. Clinical analysis of C1 and C2 subtypes indicated that C1 patients with a poor prognosis had a higher mortality, T staging, grade, and AJCC staging than those with C2 subtype, and there were no significant differences in gender, N stage, or M stage between the two subtypes (Fig. 6D).

### Risk models were constructed based on prognosis-related HRD genes

**Figure 7:**
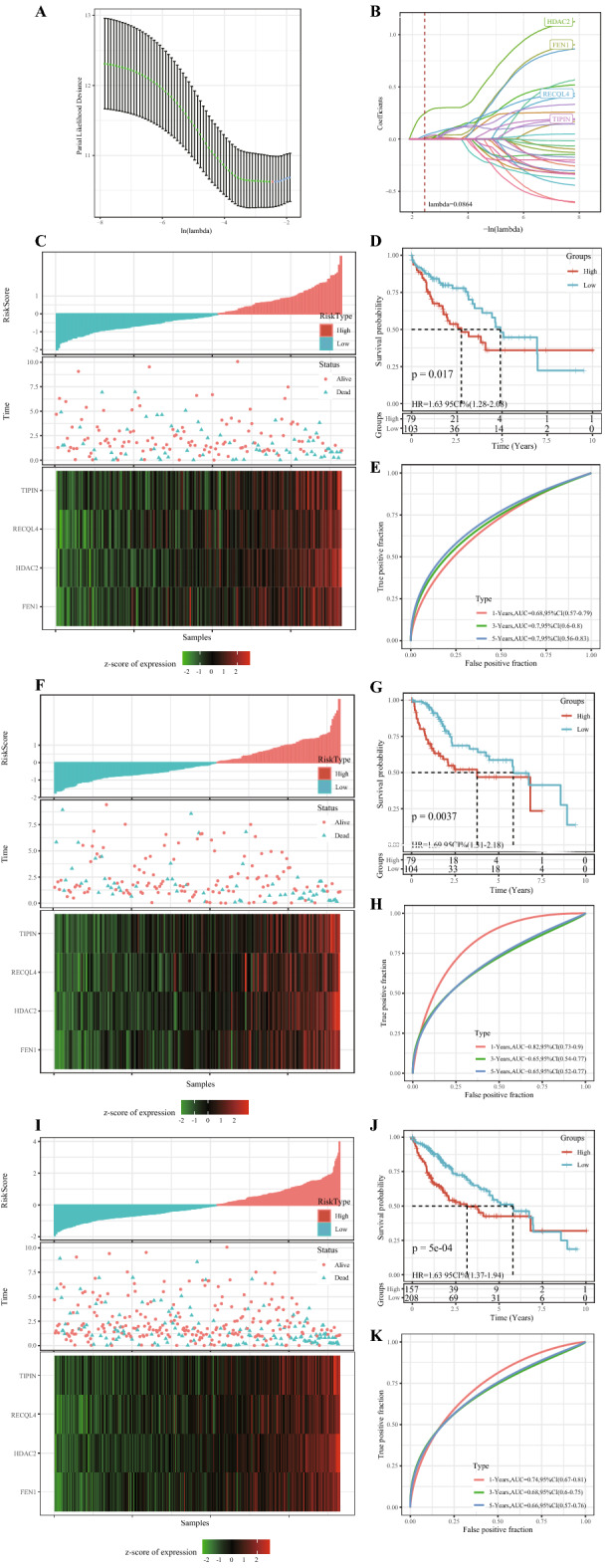
Risk models were constructed based on prognosis-related HRD genes. (**A**) Screening of optimal lambda. (**B**) LASSO coefficient spectra of 33 HCC prognostic genes. (**C**) The risk scores of the TCGA training cohort were arranged in order from low to high, the existential state of the patients and the expression heat maps of the four genes. (**D**) Kaplan–Meier survival analysis uncovered differences in OS between high-risk and low-risk patients in the TCGA training cohort. (**E**) ROC curves of 1-year, 3-year, and 5-year OS of TCGA training cohort samples. (**F**) In the TCGA validation cohort, the risk scores of the cases were arranged in order from low to high, the existential state of the cases and the expression heat maps of the four genes. (**G**) Kaplan–Meier survival analysis revealed differences in OS between high-risk and low-risk patients in the TCGA validation cohort. (**H**) 1-year, 3-year, and 5-year OS curves for patients in the TCGA validated cohort. (**I**) Risk score arrangement, survival state and heat map of expression of 4 genes for samples in the entire TCGA dataset. (**J**) Survival curves of patients at different risk across the TCGA dataset. (**K**) ROC curves of 1-year, 3-year and 5-year OS samples in the entire TCGA dataset.

Univariate Cox analysis identified 33 genes significantly associated with HCC prognosis (Supplementary Table S2). LASSO- Penalized Multivariate Cox analysis (Fig. 7A,B was performed on these 33 genes to establish a four-gene signature with the formula as follow:

$${\text{Risk}}\;{\text{score}} = 0.0{64}*{\text{FEN1}} + 0.{416}*{\text{HDAC2}} + 0.{149}*{\text{RECQL4}} + 0.{137}*{\text{TIPIN}}.$$

The risk score for each case in the TCGA training cohort was calculated and ranked from the lowest to the highest. The risk score was positively related to the number of death cases. The heat map demonstrated that the expression of four genes differed considerably between the high-risk group (n = 79) and the low-risk group (n = 103) (Fig. 7C). The 5-year OS of the high-risk and low-risk groups also showed significant differences (Fig. 7D). The area under the ROC curve (AUC) of 1, 3 and 5-year OS was 0.82, 0.65 and 0.65, respectively (Fig. 7E). In the TCGA validation cohort and the entire cohort, the risk scores of cases were calculated according to the risk scoring formula to divide the samples into high-risk and low-risk groups (Fig. 7F,I). The difference in prognosis between the two risk groups remained significant (Fig. 7G,J). The ROC curves of 1-year, 3-year and 5-year OS showed that the signature had a high OS sensitivity and predicting accuracy (Fig. 7H,K).

### The prognostic model was validated in separate cohorts

**Figure 8:**
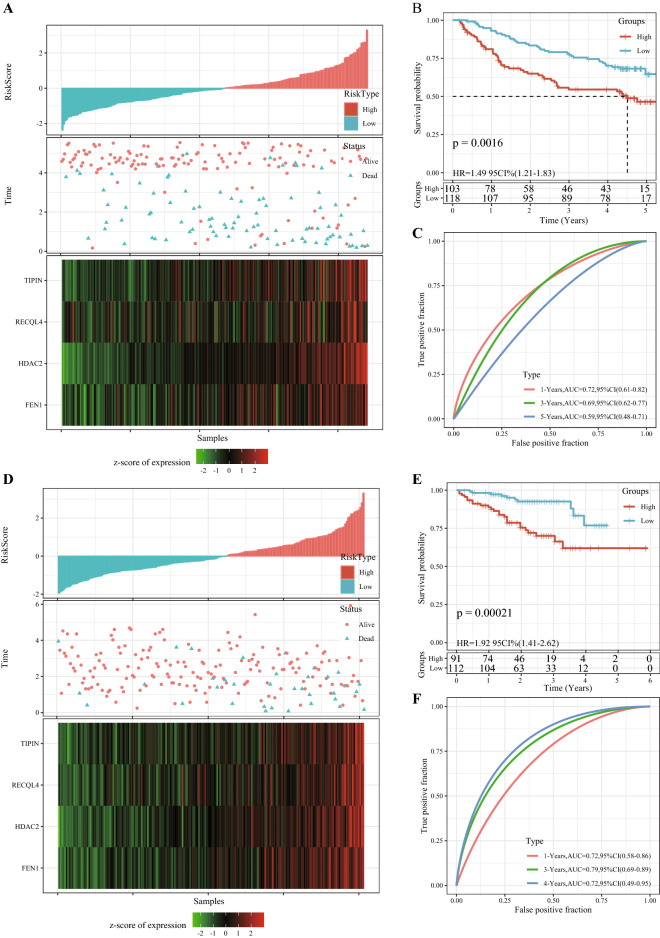
The prognostic model was validated in separate cohorts. (**A**) Risk score distribution, existential state, and 4 gene expression heat maps of HCC cases in the GSE14520 cohort. (**B**) The survival analysis of patients in different risk groups in the GSE14520 validated cohort. (**C**) 1-year, 3-year, and 5-year ROC curves in the GSE14520 cohort based on the 4-gene scoring model. (**D**) Risk score distribution, existential state and heat map of 4 genes in HCC patients in the HCCDB18 dataset. (**E**) The 4-gene scoring model was used to evaluate the OS of the samples in the HCCDB18 dataset. (**F**) 1-year, 3-year, and 5-year ROC curves for HCC patients in the HCCDB18 data sets.

We validated the robustness of the risk model in two independent external validation sets. In the GSE14520 cohort, 103 cases were assigned into the high-risk group, while 118 cases were in the low-risk group. In the HCCDB18 data set, 91 samples were in the high-risk group and 112 samples were in the low-risk group. This suggested that a higher risk score may indicate a higher death risk for HCC patients. The expression of four genes was also associated with increased risk scores in patients (Fig. 8A,D). In both cohorts, the high-risk group had distinctly shorter OS than the low- risk group (Fig. 8B,E). In the external validation set of GSE14520, the AUC for 1-year, 3-year, and 5-year OS predicted by the 4-gene signature was 0.72, 0.69, and 0.59, respectively (Fig. 8C). In the HCCDB18 validation cohort, the 4-gene signature predicted that 1-year, 3-year, and 5-year OS was 0.72, 0.79, and 0.72, respectively (Fig. 8F). Overall, the 4-gene signature showed a satisfactory prediction of the prognosis of HCC.

### The 4-gene signature was a stand-alone prognostic factor for HCC

**Figure 9:**
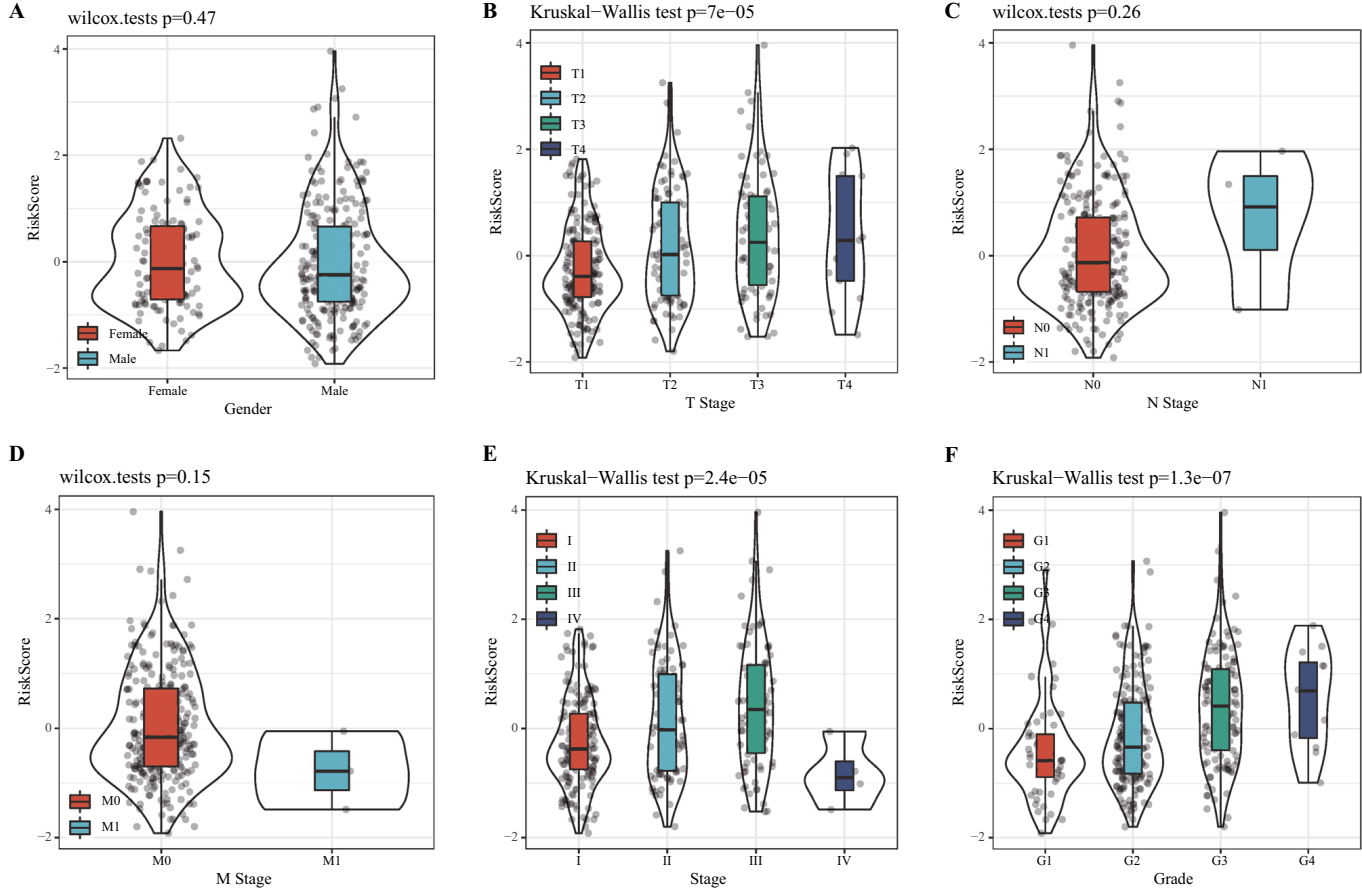
Risk score under different clinicopathological characteristics, including (**A**) gender, (**B**) T stage, (**C**) N stage, (**D**) M stage, (**E**) AJCC stage and (**F**) grade.

**Figure 10:**
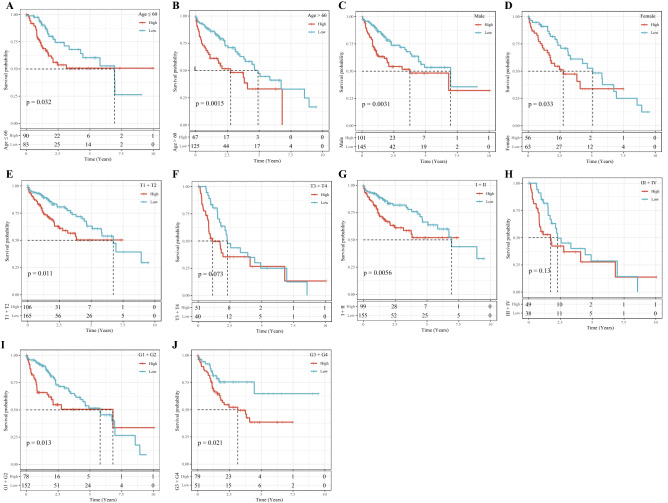
All HCC cases in the TCGA dataset were stratified according to clinicopathological parameters. (**A**) age ≤ 65; (**B**) age > 65; (**C**) Male; (**D**) Female; (**E**) T1-T2 stage; (**F**) T3-T4 stage; (**G**) stage I-II; (**H**) stage III-IV; (**I**) G1-G2; (**J**) G3-G4.

**Figure 11:**
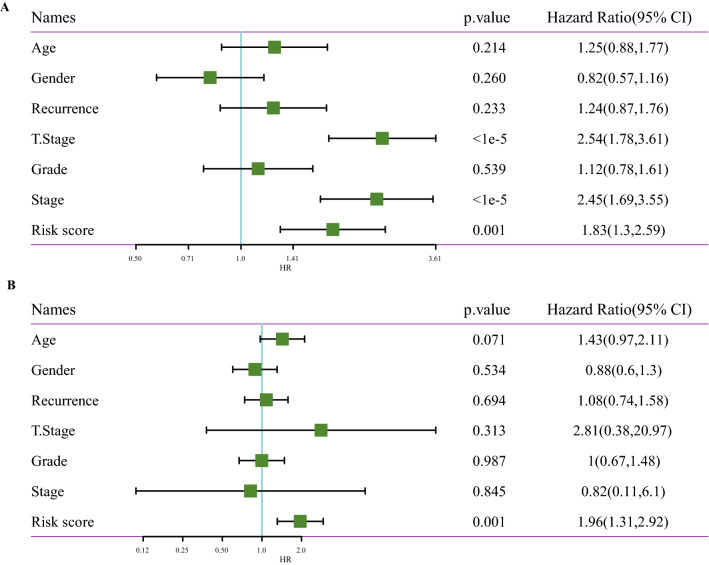
To analyze the independent prognostic value of 4-gene signature in HCC. (**A**) Univariate Cox regression analysis confirmed the association between clinicopathological factors and HCC prognosis. (**B**) Multivariate Cox regression analysis identified stand-alone prognostic factors in HCC.

Analysis on the risk scores with different clinicopathological characteristics (gender, T stage, N stage, M stage, AJCC stage and grade) showed that the risk scores of patients between male and female, N0 and N1, M0 and M1 were not significantly different. However, significant differences were detected in the risk scores among T1, T2, T3 and T4, and the risk scores increased with the increase of T stages. In addition, grade and risk score also showed an increasing trend with a higher grade. The risk scores of patients with different AJCC stages also presented significant differences (Fig. 9). Stratified analysis was performed for all cases in the TCGA dataset according to age, sex, T stage, AJCC stage and grade, and we observed that the risk scores of patients calculated by the 4-gene signature were correlated with the survival time of age ≤ 65and age > 65, male or female, T1-T2 stage or T3-T4 stage, stage I-II or stage III-IV, G1-G2 or G3-G4 (Fig. 10). The results of univariate Cox and further multivariate Cox analyses validated that risk score was an independent prognostic factor for HCC (Fig. 11A,B). These results suggested that the risk score was an accurate model for predicting prognosis of patients with HCC.

### Assessment of the risk models in predicting prognosis of HCC

**Figure 12:**
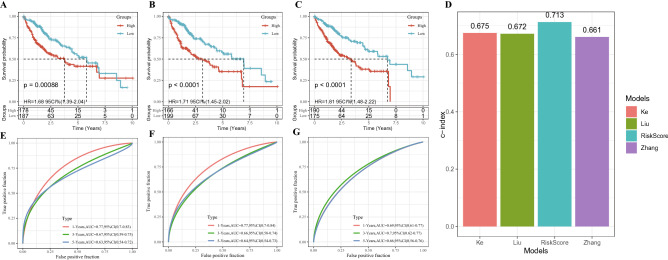
Assessment of the risk models in predicting prognosis of HCC. (**A**) The survival of samples in TCGA-LIHC was analyzed by six-gene signature (including CSE1L, CSTB, MTHFR, DAGLA, MMP10, and GYS2). (**B**) The Kaplan–Meier curve of sample survival in TCGA-LIHC was predicted by a risk model consisting of CA9, CXCL5, MMP12, SLC1A5 and G6PD. (**C**) The Kaplan–Meier curve of sample survival in TCGA-LIHC was predicted by a risk model composed of SPINK1, TXNRD1, LCAT and PZP. (**D**) C index of our risk model and the other three risk models. (**E**) ROC curve of six-gene signature for predicting HCC prognosis. (**F**) ROC curve for predicting HCC prognosis by risk model composed of CA9, CXCL5, MMP12, SLC1A5 and G6PD. (**G**) ROC curve of the model consisting of SPINK1, TXNRD1, LCAT and PZP to predict HCC survival.

To validate the reliability of the risk model in predicting prognosis, the risk model was compared with other previously reported signatures in HCC. 3 reports were selected through a small-scale literature search ^13–15^. According to the HCC prognostic model reported in each literature, the risk assessment of TCGA-LIHC samples showed that the 5-year survival rate of patients with high-risk was much worse than that of patients with low-risk (Fig. 12A-C). Figure 12D showed the C-index of each model, and the model we developed had the highest C-index. In addition, the risk model built by this study had slightly better performance in predicting long-term prognosis of HCC than the other three models (Fig. 12E-G).

## Discussion

HCC can adapt to high genomic stress conditions resulted from overactive DNA replication and genotoxic drug therapy, and the underlying mechanisms may involve enhanced DNA damage response/repair procedures^16^. Homologous recombinant DNA repair plays an important role in DNA repair. HRD has been found to be implicated in a variety of human cancers, especially in ovarian, breast, prostate, and pancreatic cancers^17^. In this study, we performed NMF clustering on HCC cases according to HRD-related genes, and identified two molecular subtypes of HCC, C1 and C2. C2 had longer OS and PFS in both subtypes, possibly because the C1 subtype was more associated with many oncology features, including mismatch repair, DNA replication, homologous recombination, cell cycle and base excision repair^18–21^. Another important reason was the large number of T cells, such as the high degree of infiltration of activated CD4 T cell, central memory CD4 T cell, type 2 T helper cell, natural killer T cell, T cells, CD8 T cells, effector memory CD4 T cell, activated memory T cells, CD4 follicular helper T cells and regulatory T cells in the C1 subtype tissues^22,23^.

Integrating various independent prognostic variables into a single formula could significantly improve prognostic prediction ability. To address the heterogeneity between and within various HCC subtypes, the use of multiple genes rather than one single gene or pathway have been applied to define the risk for HCC initiation, progression and recurrence^24^. The joint analysis of Arjun Sarathi and Ashok Palaniappan showed that there were different stage-specific genes in different AJCC stages of HCC, specifically, they identified 2 genes specific to stage I and II, 10 specific to stage III, and 35 specific to stage IV ^25^. Recently, a five-gene predictive signature was developed and highlighted potential prediction feasibility of recurrence of early-stage HCC ^26^. In our work, 3623 DEGs between C1 and C2 subtypes were identified, and a risk score was constructed using univariate Cox analysis and LASSO-Cox regression analysis. Patients were ranked according to their risk score, and the number of death cases was found to be positively related to the risk score. Subgroup analysis revealed that the model was suitable for identifying and predicting HCC patients with different clinical characteristics. More importantly, our scoring model could accurately assess the prognostic risk of HCC cases in two independent external validation cohorts. We also noted that the risk scores of patients calculated according to the 4-gene signature were significantly correlated with age ≤ 65 and age > 65, male or female, T1-T2 stage or T3-T4 stage, stage I-II or stage III-IV, G1-G2 or G3-G4, indicating that the risk scoring model had a strong applicability. Additionally, the risk score was established as a stand-alone prognostic marker for HCC. All these evidence suggested a great potential of the risk scoring model for bedside application.

To conclude, study classified two molecular subtypes of HCC with different immune-infiltrating states and clinical characteristics. In addition, we established a 4-gene signature that showed high specificity and sensitivity in evaluating the prognosis of HCC and can be used as a stand-alone prognostic factor. To the best of our knowledge, our model was the first prognostic model constructed based on these four genes, and it could facilitate personalized treatment of HCC, as the signature showed a high stability and general applicability.

## Methods

### Organization and processing of original data

Original expression profile information and clinical data of HCC downloaded were from the TCGA-LIHC, HCCDB18 (http://lifeome.net/database/hccdb/home.html) and Gene Expression Omnibus (GEO) database. For the TCGA-LIHC dataset, batch number of each sample was obtained from UCSC Xenabrowser (https://xenabrowser.net/datapages/?dataset=TCGA-LIHC.GDC_phenotype.tsv&host=https%3A%2F%2Fgdc.xenahubs.net&removeHub=https%3A%2F%2Fxena.treehouse.gi.ucsc.edu%3A443), and the combat function of R software package SVA was used for batch effect removal. The expression of genes with multiple probes in the TCGA dataset was the median value of these probes. When a probe corresponding to multiple genes in the HCCDB18 dataset, it was removed. After processing, 365, 203 and 221 HCC samples with complete clinical data from TCGA, HCCDB18 and GSE14520 were obtained. Table 1 presents the clinicopathological statistics of the samples from the 3 datasets. 108 HRD-related genes were collected from other studies^4,11,27–37^. Supplementary Fig. S1 shows the study design.

**Table 1:** Clinicopathological statistical information of HCC patients in the TCGA, HCCDB18, and GSE14520 datasets.

Clinical features	TCGA-HCC	HCCDB18	GSE14520
**OS**			
0	235	168	136
1	130	35	85
**T stage**			
T1	180		
T2	91		
T3	78		
T4	13		
TX	3		
**N stage**			
N0	248		
N1	4		
NX	113		
**M stage**			
M0	263		
M1	3		
MX	99		
**Stage**			
I	170		
II	84		
III	83		
IV	4		
X	24		
**Grade**			
G1	55		
G2	175		
G3	118		
G4	12		
GX	5		
**Gender**			
Male	246		
Female	119		
**Age**			
≤ 60	173		
> 60	192		
**Recurrence**			
YES	198		
NO	167		

### Molecular subtypes were identified by nonnegative matrix factorization (NMF) clustering

The expression of 109 HRD genes was obtained from TCGA, and univariate COX analysis was performed with coxph function in R. The HCC samples were clustered by NMF^38^. Specifically, the standard "Brunet" was used for 100 iterations. The number of clusters k was set to 2–10, the average contour width of the common member matrix was determined using R package "NMF". In addition, Kaplan–Meier curve and log-rank test were used for survival analysis.

### Difference analysis and enrichment analysis

The differences of different molecular subtypes were analyzed by R-package Limma^39^. Differentially expressed genes (DEGs) were analyzed using WebGestalt ^40^(V0.4.2) for KEGG pathway enrichment and GO function enrichment analysis. In addition, to analyze the enrichment of different molecular subtypes in different pathways, the cp.kegg.v7.0.symbols.gmt gene set was used as the reference gene set for Gene Set Enrichment Analysis (GSEA). The pathways with *P* < 0.05 and false discovery rate (FDR) < 0.25 threshold were considered as significantly enriched.

### Immune scores and clinical characteristics of HCC patients with different molecular subtypes

R software package single-sample gene set enrichment analysis (ssGSEA)^41^, MCP counter^42^, CIBERSORT^43^ were used to measure the immune score of each sample. Clinical characteristics of different molecular subtypes, including survival status, sex, TNM stage and AJCC stage, were analyzed using ggplot2^44^.

### Construction and evaluation of a prognostic scoring system

The 365 samples in the TCGA dataset were grouped into a training set (n = 182) and a verification set (n = 183). Chi-square test showed no significant differences in overall survival (OS), TNM stage, clinical stage, grade, gender or age between the training set and the validation set (Table 2). In the training set, the relationship between HRD gene and HCC was determined by univariate Cox regression analysis. Least absolute correlation and selection operator (LASSO) and multivariate Cox regression analysis were performed to filter the HRD genes significantly associated with HCC prognosis and to establish a risk scoring system. Patients were grouped into high-risk and low-risk groups based on their standardized risk scores. R software package timeROC was used to generate receiver operating characteristic curve (ROC). Finally, univariate and multivariate Cox regression analyses were conducted to evaluate the independence of the prognostic model.

**Table 2:** TCGA training set and validation set sample information table.

Clinical features	TCGA-HCC train	TCGA-HCC test	P
**OS**			
0	114	121	0.5582
1	68	62	
**T stage**			
T1	91	89	0.9113
T2	47	44	
T3	36	42	
T4	7	6	
TX	1	2	
**N stage**			
N0	120	128	0.1145
N1	4	0	
NX	58	55	
**M stage**			
M0	133	130	0.1715
M1	3	0	
MX	46	53	
**Stage**			
I	83	87	0.3301
II	43	41	
III	39	44	
IV	4	0	
X	13	11	
**Grade**			
G1	26	29	0.6808
G2	88	87	
G3	59	59	
G4	5	7	
GX	4	1	
**Gender**			
Male	125	121	0.6816
Female	57	62	
**Age**			
≤ 60	87	86	0.9604
> 60	95	97	

### Statistical analysis

The statistical analysis in this study was performed in R software, and the differences in clinicopathological and molecular features between different subtypes were calculated by student t- test and chi-square test. *P* < 0.05 was seen to be statistically significant.

## Supplementary Information

Supplementary Information 1.

Supplementary Information 2.

Supplementary Figure S1.

Supplementary Information 4.

Supplementary Information 5.

Supplementary Table S1.

Supplementary Table S2.

## Data Availability

The datasets generated and/or analysed during the current study are available in the [GSE14520] repository, [https://www.ncbi.nlm.nih.gov/geo/query/acc.cgi?acc=GSE14520].

## Code availability

The code for generating the risk score model discussed in this study is available at Supplementary code.R.
