# Supplementary material for: Identification of molecular subtypes and prognostic signature for hepatocellular carcinoma based on genes associated with homologous recombination deficiency
Source: Sci Rep. 2021 Dec 15;11:24022. doi: 10.1038/s41598-021-03432-3 (PMC8674316; doi:10.1038/s41598-021-03432-3)
Supplement: Supplementary file 1 — Supplementary Information 1. [file 41598_2021_3432_MOESM1_ESM.docx]

**Description of supplementary materials**

Supplementary Figure S1 The overall study design and workflow.

Supplementary Table S1 Differentially expressed genes between C1 and C2

Supplementary Table S2 Univariate Cox regression analysis of 33 genes significantly associated with HCC prognosis

Supplementary S1 GO terms and KEGG pathway of up-regulated DEGs between C1 and C2

Supplementary S2 GO terms and KEGG pathway of down-regulated DEGs between C1 and C2
