# Supplementary figures and images for: Identification of molecular subtypes and prognostic signature for hepatocellular carcinoma based on genes associated with homologous recombination deficiency
Source: Sci Rep. 2021 Dec 15;11:24022. doi: 10.1038/s41598-021-03432-3 (PMC8674316; doi:10.1038/s41598-021-03432-3)

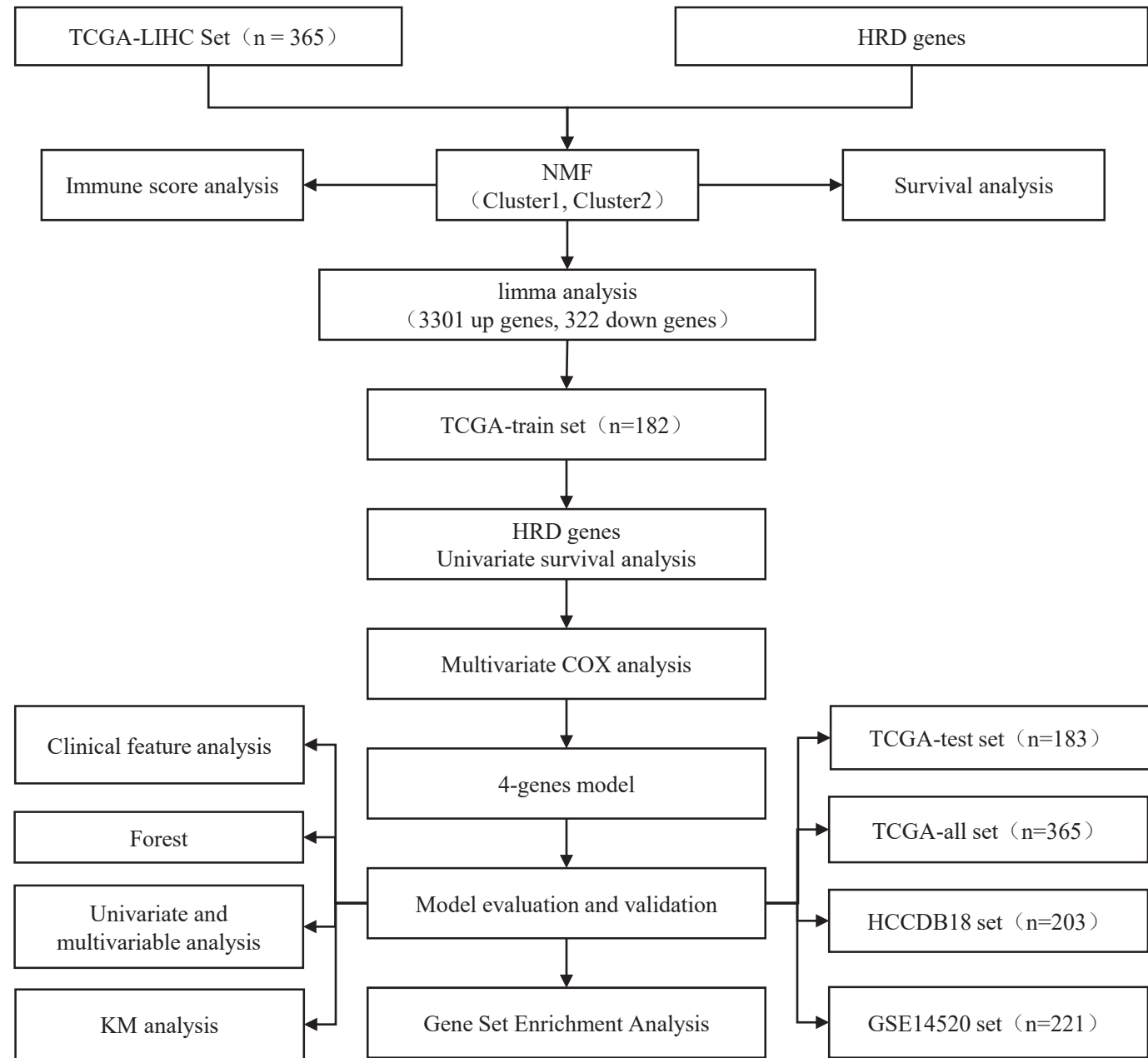

Supplement: Supplementary file 3 — Supplementary Figure S1. [file 41598_2021_3432_MOESM3_ESM.pdf]
